# Associations between youth lifestyle habits, sociodemographic characteristics, and health status with positive mental health: A gender-based analysis in a sample of Canadian postsecondary students

**DOI:** 10.1016/j.pmedr.2025.103015

**Published:** 2025-02-21

**Authors:** Rachel Surprenant, David Bezeau, Gabriel A. Tiraboschi, Gabrielle Garon-Carrier, Isabelle Cabot, Magaly Brodeur, Caroline Fitzpatrick

**Affiliations:** a Department of Preschool and Elementary School Education, Université de Sherbrooke, Sherbrooke, QC, Canada; b Cégep de Saint-Hyacinthe, Saint-Hyacinthe, QC, Canada; c Faculty of Physical Activity Sciences, Université de Sherbrooke, Sherbrooke, QC, Canada; d Department of Psychoeducation, Université de Sherbrooke, Sherbrooke, QC, Canada; e Cégep Édouard-Montpetit, Longueuil, QC, Canada; f Performa, Université de Sherbrooke, Sherbrooke, QC, Canada; g Department of Family and Emergency Medicine, Université de Sherbrooke, Sherbrooke, QC, Canada; h Department of Childhood Education, University of Johannesburg, Johannesburg, South Africa

**Keywords:** Positive mental health, Flourishing, Anxiety, Depression, Postsecondary students, Gender differences, Lifestyle

## Abstract

**Objective:**

This study aims to estimate associations between lifestyle habits, sociodemographic characteristics, health status, and positive mental health (i.e., flourishing, languishing, moderate) and anxiety and depression symptoms in postsecondary students.

**Methods:**

This cross-sectional study used a convenience sample of 2165 Canadian first-semester postsecondary students (59 % female, 41 % men). Participants reported positive mental health using the Mental Health Continuum-Short Form and completed the Hospital Anxiety and Depression Scale to screen for probable cases of anxiety and depression in the Fall of 2023. Participants reported lifestyle habits including recreational screen time (hours/day), physical activity (minutes/week), in-person social interaction (frequency/week), and homework (hours/week). Participants reported age, gender, race/ethnicity, socioeconomic status, and health status (presence of a disability or health problem).

**Results:**

Women's weekend screen time was associated with an 11 % reduction in the odds of experiencing flourishing mental health (odds ratio [OR]: 0.89, 95 % CI, 0.83–0.95), and never engaging in in-person socializing increased the odds of women experiencing languishing mental health (OR: 3.80, 95 % CI, 1.45–9.96). More frequent engagement in physical activity and homework were each associated with an increased odds of men experiencing flourishing mental health (OR: 1.00, 95 % CI, 1.00–1.00; OR: 1.03, 95 % CI, 1.00–1.05).

**Conclusions:**

These findings highlight modifiable lifestyle habits including screen time, physical activity, in-person socializing, and homework which can be leveraged for mental health promotion among postsecondary students.

## Highlights

• The mental health of postsecondary students has deteriorated in recent years.
• Screen use was associated with lower flourishing mental health in women.
• Less in-person socializing was linked to languishing mental health in women.
• Physical activity contributed to increased flourishing mental health in men.

## 1 Introduction

Studies have documented a decline in the mental health of youth and young adults (Linden and Stuart, 2020; Liu et al., 2019). During the COVID-19 pandemic, the prevalence of anxiety and depression among college students increased significantly, with anxiety rising from 19 % before the pandemic to 37 % during the pandemic, and depression increasing from 21 % to 54 % over the same period (Li et al., 2021). Among Canadians aged 15–25, suicide has become the second leading cause of death with approximately 25 % of postsecondary students (those attending colleges, universities, or institutes) reporting that they have considered suicide (Ogrodniczuk et al., 2021). However, these studies have focused on psychiatric symptoms and problems, without investigating the positive aspects of mental health, such as resilience and emotional well-being. Additionally, studies have given little attention to how lifestyle habits contribute to positive mental health.

Mental health is conceptualized as a state of subjective well-being, involving feelings of competence, productivity, and contribution to one's community (World Health Organization, 2004). In line with this definition, positive mental health is seen not just as the absence of mental illness, but also as encompassing emotional, social, and psychological well-being, existing on a continuum that ranges from flourishing to languishing mental health (Keyes, 2002). Few studies have examined their determinants in postsecondary students (Doré et al., 2016).

Consequently, identifying modifiable behaviors that promote positive mental health in postsecondary student populations is essential. Lifestyle habits refer to behaviors such as physical activity, social interactions, and academic workload, which are commonly linked to well-being among students (Chu et al., 2022; Dubuc et al., 2017). Screen time, considered a sedentary behavior (Tremblay et al., 2010), is also frequently included in the literature as part of lifestyle habits (Kowalsky et al., 2023; Lee et al., 2023). In addition to being conceptualized as a lifestyle habit by researchers, screen time is increasingly recognized as such by public health institutions in Canada (Institut national de santé publique du Québec, 2016). Research suggests that increased screen time is associated with lower well-being and reduced face-to-face social interaction among youth (Twenge and Campbell, 2018; Twenge et al., 2019). Furthermore, adolescent girls who spend more hours a day surfing the internet are more likely to develop generalized and social anxiety symptoms (Tiraboschi et al., 2023). In contrast, moderate to vigorous-intensity physical activity has been linked to higher scores in positive mental health and reduced anxiety and depressive symptoms among postsecondary students (Doré et al., 2016). Youth engagement in in-person social interaction is also associated with positive well-being (Dissing et al., 2019). This might be especially important for young adults who are becoming independent of their parents and establishing their own social support (Asher and Weeks, 2013). Finally, homework is often described by students as an important source of stress and could play a key role in their well-being (Galloway et al., 2013).

The transition to adulthood, often coinciding with postsecondary studies, represents a critical life stage for youth. Postsecondary education comes with interpersonal and intrapersonal challenges, such as relocation from established social circles, building of new relationships, increased obligations and responsibilities, and financial autonomy (Eells, 2017). As such, this life stage is often characterized by heightened vulnerability to the development of mental health problems, particularly anxiety and depression (Mortier et al., 2018). Mental health issues can, in turn, decrease academic performance and motivation, increase risks of dropout and failure, and ultimately lead to poor employment prospects due to incomplete schooling (Moghimi et al., 2023).

Mental health in postsecondary education is also influenced by students' sociodemographic characteristics, such as gender, race/ethnicity, and socioeconomic status (Wang et al., 2022). Female postsecondary students are more likely to perceive their mental health as being poor and are more frequently diagnosed with anxiety and depressive disorders (Wiens et al., 2020). Research also suggests that college students with physical and mental disabilities have a significantly higher prevalence of anxiety and depression compared to students without disabilities (Coduti et al., 2016; McLeod et al., 2019). In terms of race/ethnicity, Hispanic, Black, and Asian American college students are less likely than White students to report having received a mental health diagnosis (Liu et al., 2019). Lower rates of mental health diagnoses among racial/ethnic minorities may result from stigma, lack of culturally sensitive interventions, and language barriers, which can discourage these individuals from seeking professional help (Liu et al., 2019). Finally, low socioeconomic status, is associated with higher levels of depression, and higher levels of mental illness diagnosis (McIsaac et al., 2023).

Previous research has identified gender-based differences in the etiology and prevalence of mental health disorders in young men and women, with women having higher levels of anxiety and depression (Kuehner, 2017). Furthermore, there are documented differences in the frequency of screen time and physical activity based on gender among youth, with adolescent boys having higher screen time and greater levels of physical activity compared to girls (Sanz-Martín et al., 2022). In addition, research suggests that girls and young women may be more vulnerable to experiencing poor mental health as their screen habits differ from those of boys and young men (Fitzpatrick et al., 2023; Tiraboschi et al., 2023; Twenge and Martin, 2020). For example, girls are more likely to use screen media for socializing compared to boys (Ciarrochi et al., 2016), which may increase their exposure to cyberbullying. Furthermore, girls spend more time on social media, which can lead to unfavorable or upward social comparisons, potentially affecting their self-esteem and well-being (Abi-Jaoude et al., 2020; Appel et al., 2016). Finally, previous research has shown that men and women differ in social determinants of health (Esteban-Gonzalo et al., 2020; Pagani et al., 2020). For these reasons, associations should be investigated for men and women separately.

There is limited research offering a gender-based portrait of how lifestyle habits and sociodemographic characteristics in postsecondary students contribute to mental health in the post-pandemic context. The purpose of this study is to estimate how the lifestyle habits of young women and men (e.g., screen time, physical activity, in-person socializing, homework), sociodemographic characteristics (e.g., age, race/ethnicity, perceived socioeconomic status), and health status (e.g. disability or health problem) are associated with the probability of having flourishing, languishing, or moderate mental health, as well as the probability of developing cases of anxiety and depression.

## 2 Methods

### 2.1 Sample

This is a cross-sectional study based on a convenience sample of 3760 adolescents and young adults between the ages of 16 and 21 recruited from 13 publicly funded colleges in the province of Quebec, Canada. In Quebec, youth attend “*collèges d'enseignement général et professionnel”* (general and vocational education colleges) (CEGEPs) after high school graduation and prior to entering University. CEGEP attendance is very common (70.2 %) (Gouvernement du Québec, 2021). In the present study, only participants who indicated that they were enrolled in their first-semester were included (57.6 % of the baseline sample). Participants with missing data on age, gender, and perceived socioeconomic status were excluded from the study (5.2 % of the baseline sample), resulting in an analytical sample of 2165 participants (*n* = 1273 women and *n* = 892 men, mean age = 17.10, standard deviation = 0.54).

### 2.2 Procedure

In the Fall of 2023, participants provided informed consent and then completed either an online (79.6 %) or paper (20.4 %) questionnaire. Most students completed the questionnaire during their physical education class time and a minority completed the questionnaire at home (7.5 %). This study was approved by the ethics review boards of all participating postsecondary institutions, and all participants signed a written informed consent before completing the survey.

### 2.3 Dependent variables

Positive mental health. Positive mental health was measured using the Mental Health Continuum-Short Form (MHC-SF), which is comprised of 14 items: three items measured the frequency of emotional well-being in the past month (e.g., “feeling happy”), five measured social well-being (e.g., “feeling that people are basically good”), and six measured psychological well-being (e.g., “feeling confident to think or express your own ideas and opinions”). Participants rated each item on a 6-point Likert scale, ranging from 0 (“Never”) to 5 (“Everyday”) how often they felt this way over the last month. From participant responses to these items, a categorical mental health classification was computed (Keyes, 2005) that includes flourishing, languishing, and moderate mental health. Participants were classified as having a *flourishing* mental health when they had at least one score of 4 or 5 on the emotional well-being items, and at least six scores of 4 or 5 on the social and psychological well-being items. Participants were classified as having *languishing* mental health when they had at least one score of 0 or 1 on the emotional well-being items, and at least six scores of 0 or 1 on the social and psychological well-being items. Finally, Participants who were neither classified as flourishing nor languishing were classified as having *moderate* mental health. This scoring approach for MHC-SF subscales exhibits strong internal consistency and reliability and sex-invariance has been established (Doré et al., 2017).

Depression and anxiety. Probable cases of anxiety and depressive disorders were self-reported using the Hospital Anxiety and Depression Scale (HADS) (Zigmond and Snaith, 1983). Participants responded to 14 items relating to both disorders in the previous seven days: seven items measured probable cases of anxiety (e.g., “I feel tense or wound up”), and seven items measured probable cases of depression (e.g., “I feel slowed down”). Each item was scored on a 4-point Likert scale (0–3). A score of zero indicates the absence of symptoms, while a score of three represents the maximum. The recommended cut-off points for identifying probable cases of concern are ≥11 for anxiety and ≥ 8 for depression, respectively (Roberge et al., 2012). The French-Canadian version of the HADS (λ = 0.82–0.89) has been shown to have strong discriminant validity and reliability (Roberge et al., 2012).

### 2.4 Independent variables: lifestyle habits

Screen time. Screen time was assessed separately for weekdays and weekend days with the question: “On average, how many hours per day do you spend on screen activities for leisure (e.g., tablet, phone, computer, television) during weekdays/weekend days?” (Bado et al., 2022; Chu et al., 2022). Participants reported screen time in hours, without predefined response options.

Physical activity. Moderate to vigorous-intensity physical activity was derived from two variables reported by participants: the frequency of physical activity during their free time outside of physical education classes (in minutes per week) and the intensity level (low, moderate, or vigorous). Examples of activities were provided to help participants categorize low (slight breathlessness, e.g., walking, yoga), moderate (moderate breathlessness, e.g., light jogging, swimming), and vigorous (significant breathlessness, e.g., running, competitive sports) intensity. These responses were then used to estimate a single variable reflecting the total weekly minutes of moderate to vigorous-intensity physical activity.

In-person social interaction. Participants responded to the following question: “In general, do you participate in social activities (e.g. going to a friend's place, going out for a drink, studying with a friend at a coffee shop, engaging in physical activity with a friend)”? Participants chose from the following response options: “Never”; “Rarely (1 time/month)”; “Occasionally (1 time/week)”; and “Frequently (>2 times/week)”.

Homework*.* Homework was assessed from the following question: “How many hours per week do you spend on your studies and homework outside of class?” Participants related their homework in hours without predefined response options. Homework is part of the routine activities of students and could influence different aspects of their lives.

Sociodemographic characteristics. Participants reported their age (in years), gender (as male, female, other/prefer not to answer), and race/ethnicity (as Caucasian (White), African/Afro-American, Asian, Hispanic/Latino-American, Other). They also reported their perceived socioeconomic status, which was assessed with the following question: “How do you see your economic situation compared to other people your age”? Answer options included “Very poor”; “Poor”; “Sufficient income”; and “Affluent”. The category “Very poor” was merged with “Poor” due to the low proportion of participants in this category.

Health status. Health status was assessed from the following question: “Do you have a disability or a health problem”? Answer options included “Yes”; and “No”.

### 2.5 Data analysis

We first conducted preliminary analyses, which included descriptive statistics to identify potential outliers, assess the normality of distributions, and computed means and standard deviations. Prior to conducting the analyses, we checked the basic assumptions of multinomial regression (Tabachnick and Fidell, 2013), testing for outliers (Mahalanobis and Cook's distance) and linearity. There was no evidence of multicollinearity among the independent variables, and we confirmed the independence of observations. We used multivariable multinominal regression with *Moderate Mental Health* as the reference category to estimate associations between participants' lifestyle habits, sociodemographic characteristics and health status and their probability of flourishing or languishing compared to moderate mental health. Multivariable logistic regression was then used to estimate how these variables are associated with the odds ratios (OR) of probable cases of anxiety and depression disorders. SPSS Statistics 28 was used to conduct analyses for this study.

## 3 Results

### 3.1 Descriptive results

Table 1 reports descriptive statistics stratified by gender for all study variables. According to the categorical mental health classification, 35.8 % of women and 44.1 % of men had a flourishing mental health, 52.7 % of women and 47.8 % of men had a moderate mental health, and 11.5 % of women and 8.2 % of men had a languishing mental health (χ2 (2, *N* = 2165) = 17.210, *p* < .001). Women scored higher than men on probable cases of anxiety (41.6 % vs 14.9 %) (χ2 (1, N = 2165) = 176.302, p < .001) and depression (24.8 % vs 21.3 %) disorders (χ2 (1, N = 2165) = 3.634, *p* = .057). The chi-square test revealed a significant association between the MHC-SF scale and the HADS scale (χ^2^ (2920, *N* = 2154) = 6764.7, p < .001). Screen time during weekdays was similar for both genders (women: 4.12 hours, men: 4.43 hours). Men engaged in significantly more physical activity per week compared to women (men: 310.71 minutes; women: 160.91 minutes). When asked about in-person social interactions, 45.8 % of women and 53.2 % of men reported frequent in-person socializing. Additionally, women spent more time on homework compared to men (10.74 hours per week vs. 7.83 hours).

**Table 1:** Descriptive statistics for postsecondary students' mental health, lifestyle habits, sociodemographic characteristics, and health status in Quebec, Canada, Fall 2023.

	Women (*N* = 1273, 58.8 %)			Men (*N* = 892, 41.2 %)		
	Mean (SD)	Categorical variables (%)	Range	Mean (SD)	Categorical variables (%)	Range
*Mental Health*						
Positive Mental Health (MHC-SF), *n* = 2165						
% Flourishing mental health		35.8			44.1	
% Moderate mental health		52.7			47.8	
% Languishing mental health		11.5			8.2	
Anxiety and depression disorders, *n* = 2155						
Probable case of anxiety disorder		41.6			14.9	
Probable case of depression disorder		24.8			21.3	
*Lifestyle habits*						
Screen time, *n* = 2095						
Weekdays (hours/day)	4.1(2.4)		0–18	4.4 (2.6)		0–15
Weekend days (hours/day)	5.1 (2.5)		0–17	5.3 (2.7)		0–15
Physical activity, *n* = 2116						
Moderate to vigorous (minutes/week)	160.9 (208.3)		0–1800	310.7 (315.6)		0–1800
In-person social interaction, *n* = 2160						
Never		2.9			4.8	
Rarely (1 time/month)		12.7			11.8	
Occasionally (1 time/week)		38.6			30.1	
Frequently (>2 times/week)		45.8			53.2	
Homework (hours/week), *n* = 2071	10.7 (7.0)		0–32.5	7.83(6.2)		0–30
*Sociodemographic characteristics*						
Age, *n* = 2165	17.1 (0.5)		16–21	17.1 (0.6)		16–21
Race/Ethnicity, *n* = 1996						
Caucasian		71.8			76.6	
African, Afro-American		9.8			8.6	
Asian		4.6			3.5	
Hispanic, Latino-American		3.2			3.0	
Other		10.6			8.4	
Perceived socioeconomic status, *n* = 2165						
Very poor/poor		11.5			15.5	
Sufficient income		50			49.3	
Affluent		38.6			35.2	
*Health status*						
Disability or health problem, *n* = 2162						
No		89.9			93.4	
Yes		10.1			6.6	

Note. SD = standard deviation. MHC-SF = Mental Health Continuum-Short Form

### 3.2 Multinomial regression

Multinomial logistic regression results are presented in Table 2. For women, each additional hour of daily weekend screen time and rarely engaging in in-person socializing reduced by 11 % and 44 % the odds of experiencing flourishing mental health (OR: 0.89, 95 % confidence interval [CI], 0.83–0.95; OR: 0.56, 95 % CI, 0.35–0.90) compared to those who had moderate mental health. For men, more minutes of moderate to vigorous-intensity physical activity and each additional hour of homework were associated with flourishing mental health (OR: 1.00, 95 % CI, 1.00–1.00; OR: 1.03, 95 % CI, 1.00–1.05). Finally, perceiving oneself as very poor/poor or as having sufficient income decreased the probability of experiencing flourishing mental health by 56 % and 45 % compared to those perceiving their income as affluent, respectively (OR: 0.44, 95 % CI: 0.26–0.74; OR: 0.55, 95 % CI: 0.39–0.78).

**Table 2:** Multinomial logistic regression estimating odds of positive mental health^a^ in postsecondary students from lifestyle habits, sociodemographic characteristics, and health status in Quebec, Canada, Fall 2023.

	Reference = Moderate mental health^d^ (*N* = 1097)			
	Flourishing mental health^b^ (*N* = 849)		Languishing mental health^c^ (*N* = 219)	
	Women	Men	Women	Men
	Odds ratio (95 % CI)	Odds ratio (95 % CI)	Odds ratio (95 % CI)	Odds ratio (95 % CI)
*Lifestyle habits*				
Screen time				
Weekdays (hours/day)	1.02 (0.95, 1.09)	1.01 (0.94, 1.08)	1.07 (0.97, 1.18)	1.02 (0.89, 1.16)
Weekend days (hours/day)	0.89** (0.83, 0.95)	0.95 (0.89, 1.02)	0.95 (0.86, 1.05)	1.07 (0.95, 1.21)
Physical activity				
Moderate to vigorous (minutes/week)	1.00 (1.00, 1.00)	1.00* (1.00, 1.00)	1.00 (1.00, 1.00)	1.00 (1.00, 1.00)
In-person social interaction				
Never	0.45 (0.16, 1.30)	0.40 (0.16, 1.01)	3.80** (1.45, 9.96)	1.91 (0.66, 5.52)
Rarely (1 time/month)	0.56* (0.35, 0.90)	0.66 (0.39, 1.14)	2.14* (1.16, 3.96)	1.58 (0.66, 3.68)
Occasionally (1 time/week)	0.80 (0.60, 1.07)	0.93 (0.65, 1.34)	1.49 (0.92, 2.4)	0.91 (0.44, 1.90)
Frequently (>2 times/week)	–	–	–	–
Homework (hours/week)	1.01 (0.99, 1.02)	1.03* (1.00, 1.05)	1.00 (0.98, 1.03)	1.04 (1.00, 1.09)
*Sociodemographic characteristics*				
Age (years)	0.95 (0.69, 1.29)	0.97 (0.71, 1.31)	0.86 (0.57, 1.28)	1.22 (0.79, 1.89)
Race/Ethnicity				
Caucasian (white)	–	–	–	–
African, Afro-American	1.36 (0.83, 2.23)	0.98 (0.52, 1.86)	2.30** (1.23, 4.30)	3.02* (1.29, 7.09)
Asian	0.89 (0.45, 1.76)	0.63 (0.26, 1.56)	1.00 (0.36, 2.76)	1.84 (0.46, 7.29)
Hispanic, Latino-American	0.94 (0.42, 2.11)	1.23 (0.45, 3.37)	1.46 (0.51, 4.19)	5.71** (1.56, 20.92)
Other	1.30 (0.84, 2.02)	0.59 (0.32, 1.12)	2.04* (1.12, 3.72)	0.88 (0.30, 2.55)
Perceived socioeconomic status				
Very poor/poor	0.60 (0.36, 1.01)	0.44** (0.26, 0.74)	1.83 (0.98, 3.41)	2.56* (1.16, 5.64)
Sufficient income	0.91 (0.69, 1.20)	0.55*** (0.39, 0.78)	1.09 (0.69, 1.74)	0.76 (0.36, 1.60)
Affluent	–	–	–	–
*Health status*				
Disability or health problem				
No	–	–	–	–
Yes	1.10 (0.71, 1.71)	1.55 (0.80, 3.00)	1.56 (0.84, 2.89)	3.17* (1.24, 8.10)

Note. CI = confidence interval. * <0.05. ** <0.01. *** <0.001.

a Positive mental health was measured using the Mental Health Continuum-Short Form (MHC-SF) on a 6-point Likert scale, which comprises 14 items: three items measure the frequency of emotional well-being in the past month, five measure social well-being, and six measure psychological well-being.

b Participants were classified as having *flourishing* mental health if they had at least one score of 4 or 5 on the emotional well-being items and at least six scores of 4 or 5 on the social and psychological well-being items.

c Participants were classified as having *languishing* mental health if they had at least one score of 0 or 1 on the emotional well-being items and at least six scores of 0 or 1 on the social and psychological well-being items.

d Participants who were not classified as *flourishing* or *languishing* were classified as having moderate mental health.

Women who never and rarely engage in in-person social interaction were 3.8 and 2.14 times more likely to experience languishing mental health, respectively (OR: 3.80, 95 % CI, 1.45–9.96; OR: 2.14, 95 % CI, 1.16–3.96). Hispanic/Latino-American men, men who perceived themselves as very poor/poor, and men with a disability or health problem were 5.71, 2.56, and 3.17 times more likely to experience languishing mental health, respectively (OR: 5.71, 95 % CI, 1.56–20.92; OR: 2.56, 95 % CI, 1.16–5.64; OR: 3.17, 95 % CI, 1.24–8.10). Finally, for both genders, being African/Afro-American was associated with a 2.3 (OR: 2.30, 95 % CI, 1.23–4.30) and 3.02 (OR: 3.02, 95 % CI, 1.29–7.09) higher chance of experiencing languishing mental health.

### 3.3 Logistic regression

Logistic regression results are presented in Table 3. Women who spent more time doing homework and perceived themselves as having sufficient income were 1.04 and 1.41 times more likely to experience a probable case of anxiety disorder (OR: 1.04, 95 % CI, 1.02–1.05; OR: 1.41, 95 % CI, 1.07–1.84). For both genders, a very poor/poor perceived socioeconomic status was significantly associated with higher levels of a probable case of anxiety (women: OR: 1.85, 95 % CI, 1.20–2.87; men: OR: 2.85, 95 % CI, 1.58–5.15), as was having a disability or health problem (women: OR: 2.23, 95 % CI, 1.47–3.36; men: OR: 2.21, 95 % CI, 1.09–4.45).

**Table 3:** Multivariable logistic regression estimating odds of anxiety and depression disorders^a^ in postsecondary students from lifestyle habits, sociodemographic characteristics, and health status in Quebec, Canada, Fall 2023.

	Anxiety disorder^b^ (reference = anxiety symptoms <11)		Depression disorder^c^ (reference = depressive symptoms <8)	
	Women	Men	Women	Men
	Odds ratio (95 % CI)	Odds ratio (95 % CI)	Odds ratio (95 % CI)	Odds ratio (95 % CI)
*Lifestyle habits*				
Screen time				
Weekdays (hours/day)	1.06 (0.99, 1.13)	0.98 (0.89, 1.09)	1.12** (1.04, 1.21)	0.97 (0.89, 1.06)
Weekend days (hours/day)	0.99 (0.93, 1.05)	1.02 (0.93, 1.12)	0.95 (0.88, 1.02)	1.05 (0.97, 1.14)
Physical activity				
Moderate to vigorous (minutes/week)	1.00 (1.00, 1.00)	1.00 (1.00, 1.00)	1.00 (1.00, 1.00)	1.00 (1.00, 1.00)
In-person social interaction				
Never	1.78 (0.79, 4.00)	0.74 (0.25, 2.17)	3.85** (1.67, 8.89)	2.04 (0.92, 4.54)
Rarely (1 time/month)	0.97 (0.65, 1.50)	1.63 (0.86, 3.08)	2.80*** (1.77, 4.44)	1.37 (0.77, 2.45)
Occasionally (1 time/week)	0.94 (0.71, 1.25)	1.19 (0.73, 1.95)	1.41 (1.00, 2.00)	1.17 (0.76, 1.80)
Frequently (>2 times/week)	–	–	–	–
Homework (hours/week)	1.04*** (1.02, 1.05)	1.01 (0.98, 1.04)	1.02* (1.01, 1.04)	1.01 (0.99 1.04)
*Sociodemographic characteristics*				
Age (years)	1.03 (0.78, 1.36)	1.08 (0.76, 1.53)	1.12 (0.832, 1.52)	0.98 (0.71, 1.36)
Race/Ethnicity				
Caucasian (white)	–	–	–	–
African, Afro-American	0.68 (0.43, 1.08)	1.49 (0.73, 3.02)	1.24 (0.76, 2.05)	1.53 (0.81, 2.88)
Asian	1.42 (0.77, 2.63)	1.31 (0.43, 4.03)	2.87** (1.48, 5.38)	0.75 (0.25, 2.31)
Hispanic, Latino-American	0.53 (0.24, 1.14)	1.16 (0.33, 4.10)	0.67 (0.27, 1.65)	2.16 (0.83, 5.63)
Other	0.99 (0.65, 1.49)	1.14 (0.54, 2.43)	1.11 (0.68, 1.80)	1.47 (0.78, 2.78)
Perceived socioeconomic status				
Very poor/poor	1.85** (1.20, 2.87)	2.85*** (1.58, 5.15)	3.60*** (2.20, 5.89)	2.96*** (1.73, 5.07)
Sufficient income	1.41* (1.07, 1.84)	1.21 (0.73, 1.97)	2.11*** (1.49, 2.99)	1.51 (0.98, 2.32)
Affluent	–	–	–	–
*Health status*				
Disability or health problem				
No	–	–	–	–
Yes	2.23*** (1.47, 3.36)	2.21* (1.09, 4.45)	1.34 (0.84, 2.14)	1.96* (1.03, 3.73)

Note. CI = confidence interval. * <0.05. ** <0.01. *** <0.001.

a Probable cases of anxiety and depressive disorders were measured using the Hospital Anxiety and Depression Scale (HADS) on a 4-point Likert scale, which comprises 14 items: seven items measure probable cases of anxiety in the previous seven days, and seven items measure probable cases of depression.

b Participants were classified as having a *probable anxiety disorder* when they had a score of 11 or higher on the anxiety items.

c Participants were classified as having a *probable depression disorder* when they had a score of 8 or higher on the depression items.

Women who spent time on screens during weekdays, never or rarely engaged in in-person social interaction, devoted more time to homework, identified as Asian, and perceive themselves as having sufficient income were 1.12, 3.85, 2.80, 1.02, 2.87, and 2.11 times more likely to experience a probable case of depression disorder, respectively (OR: 1.12, 95 % CI, 1.04–1.21; OR: 3.85, 95 % CI, 1.67–8.89; OR: 2.80, 95 % CI, 1.77–4.44; OR: 1.02, 95 % CI, 1.01–1.04; OR: 2.87, 95 % CI, 1.48–5.38; OR: 2.11, 95 % CI, 1.49–2.99). For men, having a disability or health problem was associated with a probable case of depression (OR: 1.96, 95 % CI, 1.03–3.73). For both genders, perceiving oneself as very poor/poor was associated with a greater odd of having a probable case of depression (women: OR: 3.60, 95 % CI, 2.20–5.89; men: OR: 2.96, 95 % CI, 1.73–5.07).

## 4 Discussion

The present study was the first to show associations between postsecondary students' lifestyle habits, sociodemographic characteristics and health status, and mental health outcomes ranging from experiencing positive mental health to probable cases of anxiety and depression disorders. Most students reported flourishing or moderate mental health (women: 89 %; men: 92 %). Probable cases of anxiety disorders were more prevalent among women, with a greater proportion exhibiting probable cases compared to men (42 % vs 15 %). In comparison, just over 20 % of men and women reported probable cases of depression. For women, never engaging in in-person socializing was strongly associated with experiencing languishing mental health and depression. Additionally, Hispanic/Latino-American men were more likely to experience languishing mental health. For both genders, a lower perceived socioeconomic status was associated with a greater likelihood of probable cases of anxiety and depression disorders.

Research has shown that symptoms of anxiety and depression in young Canadian adults were mostly comparable before and during the pandemic (Watkins-Martin et al., 2021). Other research suggests that the COVID-19 pandemic has contributed to an increase in anxiety and depression rates among college students (Elharake et al., 2022; Li et al., 2021). To our knowledge, no study has compared the mental health of postsecondary students post-pandemic with that before the pandemic. However, when compared to a study conducted 10 years ago using the same measures of mental health, we observe a deterioration in the mental health of postsecondary students (Our findings vs. Doré et al., 2016: positive mental health: 42.0 vs 46.6 (−7 %); probable cases of anxiety symptoms: 8.5 vs 7.3 (+6 %); depressive symptoms: 5.2 vs 3.9 (+6 %)). This deterioration in mental health among postsecondary students can be partly attributed to the long-term consequences of the COVID-19 pandemic. During the pandemic, youth increased screen time and reduced in-person social interactions, which have been linked to higher levels of anxiety and depression. As such, these lifestyle changes could account for the observed decline in mental health outcomes.

Importantly, we found that women who engaged in in-person social interaction less often, were more likely to experience languishing mental health and exhibit a probable case of depression. Research has shown that in-person interactions among youth have declined during the last decade (Twenge et al., 2019). Reduced time spent on in-person social interactions could affect well-being and social skills, potentially contributing to increased loneliness. Perceived socioeconomic status also influences the mental health of postsecondary students. Both women and men who perceived their socioeconomic status as lower, were more likely to exhibit probable cases of anxiety and depression disorders. Men who perceived themselves having a low socioeconomic status were also more likely experience languishing mental health. Our findings also align with studies on mental health that reveal socioeconomic disadvantage as a major determinant of mental health issues throughout the life span (Kivimäki et al., 2020). Moreover, individuals with lower socioeconomic status are more likely to adopt unhealthy lifestyle habits, such as consuming fast food (Arslan et al., 2022). These dietary habits have been associated with increased levels of inflammation which may, in turn, contribute to poorer mental health outcomes (Elbarazi and Tikamdas, 2023).

It's interesting to note that there appears to be a gender difference in the association between homework and mental health. Women who spent more time on homework reported higher levels of probable cases of anxiety and depression, while men were more likely to experience flourishing mental health. Previous work has demonstrated that students who spend long hours on homework had greater symptoms of anxiety and depression (Yeo et al., 2020). Finally, women with higher weekday screen time were more likely to present depressive symptoms, while men engaging in more physical activity were more likely to experience flourishing mental health.

Our study is not without limitations. First, we used a community-based convenience sample, which may not be generalizable to non-first-year postsecondary students. Furthermore, data were collected in Fall 2023, a period following the pandemic, when postsecondary students may still have been engaging in screen time as a coping strategy. The cross-sectional design that does not allow us to infer the direction of the association. Self-reported measures may introduce recall bias, social desirability bias, and misclassification, particularly overestimations of physical activity (James et al., 2016). Future research could also explore how the use of counseling services may serve as a protective factor in the association between lifestyle habits, sociodemographic characteristics, and positive mental health. However, the current study also has its strengths. These include the use of a large sample. We were also able to examine a wide range of mental health indicators, including positive dimensions, and used measures with validated clinical cut-offs. Furthermore, as far as we know, there is limited research on mental health among postsecondary students following the COVID-19 pandemic.

## 5 Conclusion

The findings reveal that most postsecondary students exhibited either flourishing or moderate mental health, with only a minority experiencing languishing mental health. However, the prevalence of mental health disorders remains high, especially among young women. This study highlights modifiable behaviors for promoting mental health among postsecondary students, including low screen time, physical activity, in-person socializing, and academic engagement. It also identifies low socioeconomic status and disabilities or health problem as significant conditions affecting well-being, which should be considered in preventive intervention efforts.

## Funding

The authors acknowledge funding from the Canada Research Chair's program from the Social Sciences and Humanities Research Council and support of the Fonds de recherche du Québec and the Ministère de la Santé et des Services sociaux, as part of the Program Actions concertées du Fonds de recherche du Québec – Société et culture.

## Ethics statement and informed consent

The present research was approved by the ethics review boards of all participating postsecondary institutions. Informed consent to participate was obtained from participants. All methods were performed in accordance with relevant guidelines and regulations.

## CRediT authorship contribution statement

**Rachel Surprenant:** Writing – original draft, Methodology, Formal analysis, Conceptualization. **David Bezeau:** Writing – review & editing. **Gabriel A. Tiraboschi:** Writing – review & editing. **Gabrielle Garon-Carrier:** Writing – review & editing. **Isabelle Cabot:** Writing – review & editing. **Magaly Brodeur:** Writing – review & editing. **Caroline Fitzpatrick:** Writing – review & editing, Supervision, Conceptualization.

## Declaration of competing interest

The authors declare that they have no known competing financial interests or personal relationships that could have appeared to influence the work reported in this paper.

## Data Availability

The authors do not have permission to share data.
